# The Cost of Providing Combined Prevention and Treatment Services, Including ART, to Female Sex Workers in Burkina Faso

**DOI:** 10.1371/journal.pone.0100107

**Published:** 2014-06-20

**Authors:** Fiona Cianci, Sedona Sweeney, Issouf Konate, Nicolas Nagot, Andrea Low, Philippe Mayaud, Peter Vickerman

**Affiliations:** 1 Social and Mathematical Epidemiology Group, Department of Global Health and Development, London School of Hygiene and Tropical Medicine, London, United Kingdom; 2 UR-VIH/MA, Centre Muraz, Bobo-Dioulasso, Burkina Faso; 3 INSERM U1058 & University of Montpellier 1, Montpellier, France; 4 Department of Clinical Research, London School of Hygiene and Tropical Medicine, London, United Kingdom; 5 Department of Social and Community Medicine, University of Bristol, Bristol, United Kingdom; Alberta Provincial Laboratory for Public Health/University of Alberta, Canada

## Abstract

**Background:**

Female Sex workers (FSW) are important in driving HIV transmission in West Africa. The Yerelon clinic in Burkina Faso has provided combined preventative and therapeutic services, including anti-retroviral therapy (ART), for FSWs since 1998, with evidence suggesting it has decreased HIV prevalence and incidence in this group. No data exists on the costs of such a combined prevention and treatment intervention for FSW. This study aims to determine the mean cost of service provision per patient year for FSWs attending the Yerelon clinic, and identifies differences in costs between patient groups.

**Methods:**

Field-based retrospective cost analyses were undertaken using top-down and bottom-up costing approaches for 2010. Expenditure and service utilisation data was collated from primary sources. Patients were divided into groups according to full-time or occasional sex-work, HIV status and ART duration. Patient specific service use data was extracted. Costs were converted to 2012 US$. Sensitivity analyses considered removal of all research costs, different discount rates and use of different ART treatment regimens and follow-up schedules.

**Results:**

Using the top-down costing approach, the mean annual cost of service provision for FSWs on or off ART was US$1098 and US$882, respectively. The cost for FSWs on ART reduced by 29%, to US$781, if all research-related costs were removed and national ART monitoring guidelines were followed. The bottom-up patient-level costing showed the cost of the service varied greatly across patient groups (US$505–US$1117), primarily due to large differences in the costs of different ART regimens. HIV-negative women had the lowest annual cost at US$505.

**Conclusion:**

Whilst FSWs may require specialised services to optimise their care and hence, the public health benefits, our study shows that the cost of ART provision within a combined prevention and treatment intervention setting is comparable to providing ART to other population groups in Africa.

## Introduction

In 2011 there were 34 million people living with HIV/AIDS, with approximately two thirds in Sub-Saharan Africa [1] Anti-Retroviral Treatment (ART) has long been recognised as the mainstay therapy for HIV infected individuals, resulting in decreased mortality rates and increased life expectancy. HAART has also recently been shown to dramatically reduce the risk of HIV acquisition amongst HIV discordant couples [2]–[4], sparking heated debate regarding its use as a prevention strategy to reduce HIV transmission rates, including amongst high-risk groups such as Female Sex Workers (FSW) [5]–[7]. Early diagnosis and treatment of these individuals would not only benefit them but could also reduce HIV transmission to their clients and general population [2]. This could be especially important in concentrated epidemic settings, as seen in West Africa, where FSW are thought to drive HIV transmission [8]–[11].

This central role of FSW in HIV transmission has led to the development of numerous targeted prevention programmes, which can result in large reductions in risk behaviour and HIV prevalence amongst participants [12], [13]. The cost-effectiveness of these interventions in middle income countries has already been shown [14]–[18] However, there is limited data on interventions offering combined HIV prevention and treatment services for FSW, with evidence suggesting this marginalised group may currently be underserved [19], [20].

In Burkina Faso, the HIV prevalence amongst the general population was 1.2% in 2009 [21], and between 7.7% and 36.2% [22] amongst FSW depending on region. The Centre Muraz, a bio-medical research centre in Bobo-Dioulasso, initiated the Yerelon (“know yourself” in vernacular language) research project in 1998 to develop and test a programme of combined prevention and treatment services for FSW, including ART provision [23] The clinic is sponsored and funded by Agence Nationale de Recherche sur le Sida. (ANRS).

With this current widespread financial crisis, economic evaluations have become crucial in guiding priority setting. To date, there is no cost data available on the provision of ART to FSW. Through the collection of resource use and cost data, we aimed to estimate the overall and per patient yearly cost of providing services to FSW attending the Yerelon project. We also investigated how costs varied between different occupational exposure categories or by ART duration.

## Methods

### Study Setting

The Yerelon project relies on peer educators to recruit, educate and ensure high follow-up rates of FSW. It is a specialised clinic that provides condoms, STI testing/treatment, HIV testing and counselling, ART, medical consultations and safer sex counselling and psychological support. Peer-educators are crucial to the clinic's success as they identify FSW in the ‘milieu’, get them tested and treated at the clinic and retain them in care. They also conduct daily group education sessions with women in the waiting room. Both a pharmacy and laboratory are on site and all services are free of charge to participants.

The clinic offers a number of different visit types. As part of the research cohort, FSW are encouraged to attend the clinic every three months for a full medical visit (type “V”), which includes completion of a behavioural questionnaire, and meeting both a psychologist to discuss treatment or diagnosis issues and a doctor for a full medical examination with genital examination. The patients then undergo blood tests and microscopic examination of vaginal swabs in the laboratory, and conclude their visit at the pharmacy where a nurse dispenses any required medications. FSW also attend scheduled treatment adherence visits (type “T”) at different time intervals to receive ART medications and adherence support by a trained psychologist as required. Lastly, they can visit the clinic at any time if they are unwell to receive medical support based on their need (type “I”). Visits “T” and “I” generally do not require the psychologist, investigator or laboratory technician and are solely for medical reviews or ART dispensing. Both HIV positive and negative women follow the same clinical follow-up schedule, differing only in the laboratory investigations undertaken (Table 1).

**Table 1:** Comparison of follow-up schedule in National guidelines and at the Yerelon clinic.

Time	Vo	D15	M1	M3	M6	M9	M12
**Clinical exam**	N P B	P	NPB	NPB	NPB	N P	PB
**Psychology**	N P		N P	N P	N P	N P	N P
**Questionnaire**	N P		N P	N P	N P	N P	N P
**HIV Serology**	N PB		N	N	N	N	N
**Vaginal swab**	N PB		N P	N P	N P	N P	N P
**Syphilis** *	N PB						
**CD4 count**	N PB		P	P	PB	P	PB
**Viral load**	N P				PB		PB
**bHCG** **	N P		N P	N P	N P	N P	N P

P = HIV Positive women; N = HIV Negative women; B = National guidelines;

*VDRL test is used;

**Urinary pregnancy test; VO = First visit; D = day following enrolment; and M = month following enrolment.

Since 1998, the project has enrolled 917 women with evidence suggesting HIV incidence has reduced over this period [23]. Recently, the project has also demonstrated high adherence to ART in this marginalised group, with long-term virological and immunological responses being comparable to those in the general population [23]–[25].

### Data collection

We collected cost and output data retrospectively from the Yerelon clinic in Bobo-Dioulasso, for the 12-month period from January to December 2010. Financial and economic costs were collected from the provider perspective and classified as capital or recurrent [26]. Separate start-up costs were not included as they preceded our study by 12 years and documentation was not available. They were deemed negligible by the principal investigator Recurrent inputs included personnel, supplies and building operation and maintenance, while capital costs included building space, training and equipment. Capital costs were depreciated using the Burkina Faso Central Bank discount rate of 4.25% [26], [27]. The life span of equipment was estimated from the German Government Technical Agency [28] and by interviewing staff.

Data on patient numbers and total visits were extracted from the project's database. Pricing information was gathered from pharmacy and equipment catalogues and project accounts. Prices for anti-retroviral drugs were estimated using clinic receipts. All costs were collected in West African Francs (CFA), inflated to 2012 prices where appropriate (CPI 2.8%) [29], and converted to US$ using the 2012 average exchange rate (1US$ = 499.51 CFA) [30].

Firstly, a top-down approach [31] was used to calculate the total annual cost of running the clinic. Top-down costs included all costs required to deliver health services within the Yerelon clinic, and were estimated from clinic administrative records. Data on consumption of consumables such as small laboratory items including reagents, slides and swabs was not available. Thus, receipts of consumable purchases during the study period were used. All anti-retroviral drugs and female condoms were donated goods and were consequently considered as economic costs and were priced according to central pharmacy price lists. Administration costs charged to the Yerelon project by Centre Muraz were 10% of the clinic's budget and were included in building operation costs. Centre Muraz laboratory undertook all haematology and biochemistry investigations, and itemised bills to the Yerelon project were used to estimate costs.

Secondly, to estimate the costs of treating different patient types, we adopted a combined bottom-up (patient-level) and top-down approach [31]. We used an ingredients-based approach to estimate the cost per client, based on observed resource use. Staff time allocation was estimated per visit type (V, I, T) through staff interviews. Data on frequency of visits and consumption of drugs, supplies and lab tests were collected from individual patient records and clinic stock records. All patient details were anonymized and de-identified prior to analysis. The Ethics Committee at the London School of Hygiene and Tropical Medicine approved the study.

### Cost analysis

For the top-down analysis, we separated patients into ART and non-ART groups, with ART costs only being applied to the women on ART but all other costs being apportioned evenly across all women. We then calculated the mean annual cost per woman treated in each group, and mean cost per visit as the main output measures. This costing methodology is likely to underestimate the difference in cost between women on and off ART with the costs per women on ART likely to be higher than our estimate. However, it gives good estimates of total annual cost of the project and proportion of costs for each cost category.

For the combined top-down/bottom-up analysis, we used information from the project database to categorise women into six groups to explore differences in costs between occupational exposures and ART duration. The groups were: 1) Full-time sex workers (FTSW) and 2) Occasional sex workers (OSW), both on ART; 3) HIV Negative women; 4) Women in pre-ART care; 5) Women commenced on ART in the last year; and 6) Women on long-term ART (started ART in or before 2006).

A sample of 40 women was then randomly selected [32] from facility records, with 10 selected from groups 1 and 2, and 5 selected from each other group. Unit prices were applied to all direct resources used by each patient over the relevant time period. The total cost of direct staff time for each type of visit (V, I, or T) was calculated and applied accordingly to each analysed visit. The cost of staff with no patient contact (such as security personnel) was divided equally across all visits. Indirect and overhead costs, such as rent and electricity, were estimated per patient visit using a top-down approach, and were added to direct patient costs to produce an estimated average cost of treating a woman from each group. All 30 sampled women on ART (groups 1, 2, 5 and 6) were then combined to obtain an average bottom-up cost estimate for treating one woman on ART. The data was also reanalyzed using the overlap between groups of occupational exposure and length of treatment to increase sample size.

As the only mutually exclusive groups were HIV negative, HIV positive on ART and HIV positive not on ART, the total annual cost of running the clinic was estimated by multiplying the average cost per woman in each of these groups by the total number of women in that group in 2010.

### Sensitivity analysis

A one-way sensitivity analysis was performed on the top-down estimated mean cost per woman on ART, exploring the effects of different discount rates (1%, 3% and 10%); changing all stavudine-containing (dT4) regimens to ones containing zidovudine (AZT) or tenofovir (TDF); basing the laboratory follow-up schedule on National Guidelines and reducing consumable costs by 10% or 20%. Further sensitivity analyses considered removing all research-related costs such as additional visits, laboratory investigations and staff used for research purposes and using minimum staff and equipment required to operate the clinic for therapeutic purposes alone. The high staff wages were also replaced with public sector wages [33] to approximate a national therapeutic setting. Lastly, a ‘Best Case Scenario’ was developed where research costs were removed and National follow-up Guidelines with public system wages were used.

## Results

### Total outputs

In total, 305 women attended the clinic in 2010, of which 187 women were on ART by December 2010. Of these women on ART, 29 commenced treatment during the study period. The total number of visits was 3027. A total of 40 patient records (13% of all patients), detailing 449 visits (15% of all patient visits) were analysed. Of the 30 sampled women on ART, 10 were on Zidovudine/Lamivudine/Efavirenz (AZT/3TC/EFV). Three of the women were on second line therapy Abacavir/Lamivudine/Lopinavir/ritonavir (ABC/3TC/LPV/r) and one was on alternative therapy for HIV-2 Zidovudine/Lamivudine/Idinavir/Ritonavir (3TC/AZT/IDV/RTV). The remaining women sampled were either on Triomune (3TC/d4T+nevirapine (NVP)). or Avocomb® (3TC/AZT/)+NVP.

### Total Annual Cost

The total annual financial cost of running the clinic was US$ 261,140 (Table 2). The economic cost was US$ 309,165, representing the additional value of donated buildings and ARVs. Personnel costs accounted for the biggest proportion (36.4% or US$ 112,621) of economic costs, followed by building operations (17%) and laboratory costs (16%). ARVs only accounted for 13% of total costs (US$ 40,500).

**Table 2:** Top-down estimated financial and economic costs of running the clinic in 2010 (in 2012 US$).

Cost Category	Financial US$	Total %	Economic US$	Total %
Capital	9,025	3.5	20,611	6.7
Operations	53,592	20.9	53,779	17.4
Personnel	112,621	43.9	112,621	36.4
Transport	8,006	3.1	8,006	2.6
Medications	8,105	3.2	8,105	2.6
Consumables	12,465	4.9	12,465	4.0
Condoms	1,174	0.5	1612	0.5
Laboratory	49,968	19.5	49,968	16.2
Prophylaxis	1,499	0.6	1,499	0.5
ART			40,500	13.1
**Total**	**256,454**	**100**	**309,165**	**100**

### Unit Costs

The average economic cost across the overall cohort (ART or no-ART) was US$1,014 per patient year and US$104 per visit. This increased to US$1,098 per patient year (US$111 per visit) for individuals on ART, and decreased to US$882 per patient year (US$89 per visit) if not on ART.

Using the bottom-up approach, the average cost of a woman on ART was US$913 per patient year. When looking across different patient groups, the average annual cost of treating women on established ART therapy was the highest at US$1,117. Despite the more frequent visit schedule, the average cost of treating women just initiating ART was a third less (US$734 per year – Table 3) because of the cheaper first-line drugs used. Care for HIV negative women or those in the pre-ART group incurred the lowest annual cost at US$505 and US$545, respectively. As well as not being on ART these women also attended 68% fewer visits, thus incurring less cost.

**Table 3:** Average category cost in each patient group and for overall bottom-up and top-down costing.

	Patient sub-group								
Cost Category	HIV-negative	HIV-positive not on ART	Started ART in 2010	Established ART	FTSW on ART	OSW on ART	Bottom-up on ART	Top- down on ART	
Personnel									
* Direct*	47.7	32.5	59.3	55.5	50.4	55.1	55.1	314.8	
* Non-direct*	54.5	54.5	54.5	54.5	54.5	54.5	54.5	54.5	
ART			121.3	515.4	263.8	373	318.4	216.6	
Prophylaxis		3.8	8.7	7.6	8.3	10.8	8.9	4.9	
Medications	12.5	10.8	16.7	13.7	18.4	11.5	15.1	26.6	
Laboratory	69.6	127.4	155.2	156.1	102.4	149.6	140.8	163.8	
Peer-educators*									
Condoms	9.2	4.3	6.8	2.9	21.5	5.8	9.3	5.3	
Overhead	311.1	311.1	311.1	311.1	311.1	311.1	311.1	311.1	
Mean/patient	504.6	544.8	733.5	1116.8	830.4	971.3	913	1098.08	
Range	468.2–571.1	500.6–591.7	706.9–775.2	675.1–2045.5	681.8–1196.7	694.8–1776.1			
Mean/visit	56.7	70	68	99.4	76.7	78.3	80.6		

OSW denotes occasional sex worker; and FTSW denotes full time sex worker

*In charge of patients seeking, education/behaviour session, patient tracking and involved in ART adherence support along with the psychologist, pharmacy nurse and physician.

Treatment costs for OSWs were higher than those for FTSWs because two OSW were on more expensive anti-retroviral therapies (second-line therapy or AZT/3TC/IDV/RTV), whereas one FTSW died and two others dropped out of the programme for 5–7 months. The cost per visit across patient groups ranged from US$57 to US$99 depending on staff time encountered, laboratory tests performed and whether ARTs were given. Using the overlap between groups increased sample sizes by 10% (FTSW) to 160% (OSW), but had little effect on the results obtained.

A major proportion of costs across all groups were overhead costs of US$356 for each patient, of which US$55 were non-direct staff costs. ART costs differed greatly between patients, depending on the regimen being taken. Second-line therapy (ABC/3TC/LPV/r) was three times more expensive (US$1356 per patient year) than the most commonly prescribed first-line regimen AZT/3TC/EFV (US$394 per patient year). The total costs of non-ART medication were low across all patient groups (12%–17% of total cost). Laboratory costs were stable across all patient groups, except amongst the HIV-negative women who had HIV serology testing but no viral load and CD4 cell count tests.

When using the bottom-up or patient-specific data, the total annual cost of the program was estimated to be US$ 237,378, 23% less than the top-down approach. Discrepancies included total ART costs being 58% higher in the bottom-up calculations and personnel costs being 72% less than top-down estimates. These variations were also evident in the breakdown of the unit costs (Table 3). In the bottom-up approach, staff time was generally allocated according to patient contact time, but did not account for staff down time, or other staff activities such as monitoring and evaluation, resulting in only 55% of total staff time being allocated in the bottom-up approach. Laboratory costs were also estimated to be 23% lower than the top-down estimates.

### Sensitivity Analysis

All sensitivity analyses (Table 4) were performed on the top-down cost estimates per patient year on ART. Firstly, varying the discount rates (1%, 3% or 10%) had very little effect (<1%), as did decreasing consumption of consumables (by 10% or 20%). Similarly, switching all patients on d4T regimens to AZT regimens, as per WHO guidelines [34], also had a small effect (<1%), whereas changing d4T to TDF increased the average cost by 12% (US$1,236). Changing AZT/3TC/NVP or EFV to TDF containing regimens, to decrease toxicity and improve outcomes [35], increased total ART costs for the clinic by 42% and the unit cost of a person on ART to US$1,407 per year. We also estimated the cost of increased capacity to include treatment of 500 additional women while assuming the same proportions of women on and off ART. This decreased the treatment costs to US$859 per year for women on ART and US$643 for those not on ART.

**Table 4:** Results of sensitivity analysis on mean cost of care per woman treated in 2012$.

	ART women	Non-ART women	All women
Base case	1098	882	1014
*Costs of consumables*			
−10%	1094	877	1010
−20%	1090	873	1005
Remove research costs	928	712	845
Assume national wages for staff	1040	824	956
National guidelines for laboratory testing	1029	812	945
Best case (combine 3 above)	781	564	697
Remove research cost and increase of 500 women			
Use AZT instead of d4t	1107	882	1019
Use TDF instead of d4T	1236	882	1098
Use TDF instead of d4t and AZT	1407	882	1203
*Discount rate (3% in base case)*			
1%	1095	879	1012
2%	1097	881	1013
10%	1104	887	1020

When laboratory investigations and visits were restricted to those recommended in the National Guidelines, the mean cost per patient year on ART reduced to US$1,029 (6% reduction). It also fell to US$1,040 if staff salaries were adjusted to public health sector levels. If we adjusted both staff salaries and reduced the number of staff to what would be needed if no research was undertaken, personnel costs reduced by 59%. The mean cost per patient-year on ART was US$928 when all research costs including behavioural survey visits, extra laboratory tests, clinical visits, staff and equipment necessary for these activities were excluded. In our best case scenario, when all research costs as above were excluded and remaining staff salaries were reduced to public sector levels, the mean cost per patient on ART was US$781 (29% reduction) per year.

## Discussion

The cost-effectiveness of HIV prevention programmes aimed at FSW has already been established [14]–[16], as has the cost-effectiveness of providing HAART to HIV infected individuals in resource poor settings [36], [37]. However, little is known about the cost of providing a combined intervention package, including peer-based HIV and STI prevention, counseling and ART to FSW in the same setting. Our study provides the first comprehensive costing of such an intervention- the Yerelon clinic in Burkina Faso. We found that the average annual cost for an HIV-positive FSW on ART, including research costs, was US$1,098, whereas it was US$882 for a FSW not on ART.

A large proportion of costs were attributable to personnel (36%) because the project employed 8 clinical staff and 17 support staff with higher wages than their counterparts in the public system. Laboratory costs exceeded ART costs because of elevated research laboratory costs and the high proportion of women on cheaper d4T containing therapy. The cost for non-ART medications was exceptionally low (3.5% of total); reflecting the low unit cost of generic medicines prescribed in this outpatient setting.

Analysis of bottom-up costs revealed that the difference in ART regimens was the main driver of difference in costs between groups. The average yearly cost of ART between groups ranged from US$121 amongst women recently commenced on ART up to US$515 amongst women on long established therapy. This latter group was found to have the highest overall annual cost because three women were on expensive ARV regimens costing more than US$1,356 per year. In HIV negative women, the cost of care ranged between US$468-571, largely made up of high overhead costs. The high proportion of personnel costs (20%) in this group reflects the psychological support offered with every serology test.

We found discrepancies (23%) between our two costing methods. This is not unusual as the literature reports 15–25% as a modest discrepancy [38]. The large discrepancy in personnel costs may mean the clinic was not working at full capacity (related to a reduced research funding at the time), whereas discrepancies in other cost categories may be due to heterogeneity within the cohort and our samples. As our main output measure was the mean cost per patient year, a larger sample may be required to reduce the uncertainty in our estimates, especially for ART costs where the bottom-up mean ART cost was 53% greater than the top-down estimate. The smaller discordance in laboratory costs was probably due to the lower rates of viral load testing recorded in our patient-specific samples than in our whole cohort.

Our sensitivity analysis showed that neither the discount rate used nor the uncertainty in consumables had any real impact on average costs. However, swapping existing ART regimens to ones containing tenofovir increased the mean cost per patient year by 13% for d4T regimens and 28% for AZT regimens. This has important policy implications, because as evidence mounts to support the use of tenofovir as a first line agent [35], [39] its higher cost may hamper the achievement of universal coverage. The increasing number of patients on more costly second-line therapies may also hinder universal coverage [40], [41]. It is therefore essential that low-income countries begin to explore ways to acquire these more expensive drugs at lower costs.

The clinic's research activities required both a greater number of staff and a higher number of laboratory investigations being performed. Decreasing the number of laboratory tests in line with National Guidelines resulted in a 6% reduction in average cost, whereas when all research costs were removed there was a 16% reduction in average patient cost. When we combine this with a reduction in salaries to national levels then a 29% reduction in the yearly cost per patient was achieved, giving an average cost of US$780 per patient year on ART. This highlights the heavy research costs incurred by the Yerelon clinic and suggests that integration into the public health system might be achieved at reasonable cost. Though these results are promising, they should be interpreted with caution. A reduction in resources used could result in a concurrent reduction in intervention effectiveness, possibly through lower adherence or higher loss to follow up and treatment failure from having a reduced diagnostic schedule and lower paid, less qualified staff.

Our cost estimates vary somewhat from the existing literature on HIV treatment costs; however, all comparative literature (Table 5) has examined costs of HIV treatment in the general adult population and not FSWs. Existing studies from Africa have found a large variation in costs with most being lower than ours but many having higher ART costs. The difference between our estimated annual cost and these published estimates can probably be explained by two main factors. Firstly, the Yerelon clinic incurred large research-related personnel and laboratory costs described above. When these elevated costs are adjusted, the costs from our analysis reduce below many of the estimates included in Table 5. Secondly, the intervention provided at the clinic is both preventative and therapeutic, whereas the studies in Table 5 focused solely on HIV treatment.

**Table 5:** Comparing costs of HIV treatment and care across similar studies in sub-Saharan Africa.

Study (year of costing)	Country	HIV Prevalence % [44] (year)	Cost/person year US$	Cost/person year 2012 US$ [45]
Our Study (2012)	Burkina Faso	1.2 (2009)	1098	1098/
Harling et al, 2007 [46] (2004)	South Africa	17.8 (2009)	432**	524
Houton et al, 2008 [43] (2006)	Benin	1.2 (2009)	1160	1318
Rosen et al, 2008 [39] (2006)	South Africa	17.8 (2009)	928 (756–1156)	1055(859–1314)
Menzies et al, 2011 [47] (2009)	Ethiopia Nigeria Uganda	2.1 (2007) 3.6 (2009) 6.5 (2009)	682 988 843	728 1055 900
Jaffar et al, 2009 [48] (2008)	Uganda	6.5 (2009)	834	887
Bikilla et al, 2009 [49] (2006)	Ethiopia	2.1 (2007)	269	326
Degahye et al, 2006 [50] (2004)	South Africa	17.8 (2009)	739–1073*	962–1301
Martinson et al 2009 [51] (2004)	South Africa	17.8 (2009)	1177	1428
Tagar et al 2012 [52]	Malawi Ethiopia Rwanda Zambia South Africa	10.8 (2012) 2.1 (2007) 2.9 (2012) 12.7 (2012) 17.8 (2009)		136 186 232 278 682

*converted from Rand **result given per month.

There are several limitations to our study. A provider perspective can underestimate total economic costs. In addition, we chose not to separate prevention and treatment cost as the aim of the clinic was to provide both services in a combined package. Start-up costs were also not as they were not available. These could be significant when integration into the public system occurs. Also, though the major research costs were removed in our sensitivity analysis, it was difficult to truly separate out all resources used for research purposes. Lastly, our patient-specific sample was small, representing only 13% of our cohort – this resulted in a few patients skewing the results for specific patient groups. However, though the sample is small, it does allow to examine cost differences between different exposure levels.

In this current economic climate, we must focus our limited resources in innovative ways. The pivotal role of FSW in the transmission of HIV in many settings demands a targeted programme of combined prevention and treatment for these women. Such a dedicated service, as the Yerelon clinic, can produce good results in terms of behaviour change, virological suppression and decreases in HIV incidence and prevalence, at costs which does not differ from those of routine HIV care clinics for the general population [23], [24]. Our study is the first to report on the costs of such a combined intervention for FSWs. Even at a time of severe budgetary constraints, investing in “difficult to reach”, high-risk groups should be a priority in countries with modest HIV prevalence in the general population (mainly West/North Africa and South-East Asia regions), because of the large public health gains in controlling the HIV dynamics. In addition, our study shows that the cost of this combined approach is comparable to other treatment-only interventions in the general population. This, coupled with the good biological and behavioural results seen in our cohort, suggests that this intervention may be cost-effective. The cost of this important intervention should, therefore, not be a prohibitive factor in scaling it up across Burkina Faso and elsewhere with the average costs likely to decrease as this is done [42], [43]. Of priority now, is combining impact evaluation to our cost data to determine the cost-effectiveness of such an intervention.
